# Dynamic probability integration for electroencephalography-based rapid serial visual presentation performance enhancement: Application in nighttime vehicle detection

**DOI:** 10.3389/fncom.2022.1006361

**Published:** 2022-10-14

**Authors:** Yujie Cui, Songyun Xie, Xinzhou Xie, Xiaowei Zhang, Xianghui Liu

**Affiliations:** NPUTUB Joint Laboratory of Neural Informatics, School of Electronics and Information, Northwestern Polytechnical University, Xi’an, China

**Keywords:** brain-computer interface, D–S evidence theory, electroencephalogram, human-computer fusion, rapid serial visual presentation

## Abstract

**Background:**

Rapid serial visual presentation (RSVP) has become a popular target detection method by decoding electroencephalography (EEG) signals, owing to its sensitivity and effectiveness. Most current research on EEG-based RSVP tasks focused on feature extraction algorithms developed to deal with the non-stationarity and low signal-to-noise ratio (SNR) of EEG signals. However, these algorithms cannot handle the problem of no event-related potentials (ERP) component or miniature ERP components caused by the attention lapses of human vision in abnormal conditions. The fusion of human-computer vision can obtain complementary information, making it a promising way to become an efficient and general way to detect objects, especially in attention lapses.

**Methods:**

Dynamic probability integration (DPI) was proposed in this study to fuse human vision and computer vision. A novel basic probability assignment (BPA) method was included, which can fully consider the classification capabilities of different heterogeneous information sources for targets and non-targets and constructs the detection performance model for the weight generation based on classification capabilities. Furthermore, a spatial-temporal hybrid common spatial pattern-principal component analysis (STHCP) algorithm was designed to decode EEG signals in the RSVP task. It is a simple and effective method of distinguishing target and non-target using spatial-temporal features.

**Results:**

A nighttime vehicle detection based on the RSVP task was performed to evaluate the performance of DPI and STHCP, which is one of the conditions of attention lapses because of its decrease in visual information. The average AUC of DPI was 0.912 ± 0.041 and increased by 11.5, 5.2, 3.4, and 1.7% compared with human vision, computer vision, naive Bayesian fusion, and dynamic belief fusion (DBF), respectively. A higher average balanced accuracy of 0.845 ± 0.052 was also achieved using DPI, representing that DPI has the balanced detection capacity of target and non-target. Moreover, STHCP obtained the highest AUC of 0.818 ± 0.06 compared with the other two baseline methods and increased by 15.4 and 23.4%.

**Conclusion:**

Experimental results indicated that the average AUC and balanced accuracy of the proposed fusion method were higher than individual detection methods used for fusion, as well as two excellent fusion methods. It is a promising way to improve detection performance in RSVP tasks, even in abnormal conditions.

## Introduction

Brain-Computer Interface (BCI) analyses the individual’s intentions to interact directly with devices or external environments (Wolpaw et al., 2000). Individuals’ intentions can be decoded using electroencephalography (EEG), a well-established non-invasive technology owing to its high temporal resolution, reliability, affordability, and portability. Currently, BCI has been applied in assistive and clinical fields due to the development of machine learning and deep learning methods.

Rapid serial visual presentation (RSVP) is the process of sequentially displaying images at high presentation rates of multiple images per second at the same spatial location. RSVP-based brain-computer interface (BCI) is a specific type of BCI system (Marathe et al., 2016; Wu et al., 2018). It is proven to be a realizable approach to enhance human-machine symbiosis and human potential (Manor et al., 2016). RSVP-based BCI is the most commonly used technology for target detection based on human vision, in which used event-related potentials (ERPs) are P300 and N200 (Wei et al., 2022). The human visual system is an amazingly complicated information processing machine. Humans have great learning, cognitive ability, and sensitivity, which can identify objects at a glance (Sajda et al., 2010). Therefore, RSVP-based BCI can obtain a rapid perception of the environment owing to the flexible human vision.

The current research focused extensively on proposing more reliable and effective feature extraction algorithms suitable for the RSVP-based BCI. Because of its non-stationarity and low signal-to-noise ratio (SNR), it is difficult to distinguish target and non-target stimuli in the RSVP task. Sajda et al. (2010) developed an algorithm named hierarchical discriminant component analysis (HDCA), which adopts fisher linear discrimination (FLD) to calculate weights in the spatial domain and then adopts Logistic Regression (LR) to calculate weights in the time domain. Alpert et al. (2014) proposed a spatially weighted FLD-PCA (SWFP) method which uses principal component analysis (PCA) and FLD to extract temporal and spatial features. They contrasted the performance of SWFP and HDCA, and the areas under receiver operating characteristic (ROC) curves (AUC) of SWFP are higher than HDCA (Alpert et al., 2014). Xie et al. (2022) used a filter bank spatial-temporal component analysis (FBSCA) algorithm, and this approach obtains spatial-temporal features from the gamma-band. Xiao et al. (2021) developed a discriminative canonical pattern matching approach, a robust classifier for ERP components assessing even in small training sets.

Most of the research focused on improving the performance of the RSVP-based BCI by exploring a more robust and effective algorithm. However, the performance of the RSVP-based BCI relies not only on superior algorithms, but also on human vision. Human vision is limited by factors that affect cognitive levels, such as fatigue, boredom, heavy mental workload (Lee et al., 2016), and some abnormal conditions, such as the decrease in visual information and the complex illumination environment. These may also lead to attention lapses representing no ERP component or miniature ERP components in the target state. It is difficult for traditional algorithms to solve these types of problems. Although the credibility and stability of computer vision are unchangeable over time, computer vision systems are not powerful enough to handle all possible situations. Considering that the fusion of human vision and computer vision can obtain complementary information, thus it may be a promising way to become an efficient and general detection method.

Some researchers have reported methods that fuse computer vision and human vision. Sajda et al. (2010) proposed three basic modes for creating cortically coupled computer vision systems, including computer vision followed by EEG-RSVP, EEG-RSVP followed by computer vision, and tight coupling of EEG-RSVP and computer vision (Gerson et al., 2005). On this basis, Pohlmeyer et al. (2011) proposed a close-loop C3Vision system to help a user in finding their “interest” images through a large database. Their research mainly explores how to expedite the search for large image databases and even identify the user’s intent (Pohlmeyer et al., 2011). Based on the research of Sajda et al. (2010), we explored the human-computer fusion framework under their third mode, which is the tight coupling of EEG-RSVP and computer vision. Unlike the study of Pohlmeyer et al. (2011), we aim to explore how to fuse computer vision and human vision to modify the classification performance, even in abnormal conditions.

Computer vision and human vision are heterogeneous information sources with different data models, interfaces, schemes, and representations (Motro and Anokhin, 2006). The fusion methods for heterogeneous information sources can be divided into two categories: (i) feature level (early fusion) and (ii) higher semantic level (late fusion) (Jaimes and Sebe, 2007). Many feature-level fusion methods are quickly becoming obsolete because they are limited to the types and internal structures of the features (Wong et al., 2007). Late fusion can maintain a balance between the amount of feature information and the difficulty of information procession, regardless of data type and data processing. Therefore, late fusion is the most popular fusion method for heterogeneous information sources (Xiao et al., 2022). Many types of late fusion architectures have been developed, such as naive Bayesian fusion (NBF), Dempster–Shafer (D–S) evidence theory, and weighted averages.

Kim and Ko (2005) proposed the Bayesian fusion of confidence for speech recognition. Markovic and Petrovic (2014) proposed the Bayesian sensors fusion for dynamic object tracking. This method improved the estimation accuracy and is credible and robust (Markovic and Petrovic, 2014). However, the Bayesian fusion approach cannot measure the level of uncertain information, which may lead to performance degradation (Lee et al., 2016). The D–S evidence theory achieves collaborative reasoning by fusing heterogeneous information from multiple sources; thus, it is an appropriate framework for dealing with incomplete uncertain information (Deng et al., 2016; Jiang and Zhan, 2017; Yang and Xu, 2002). At present, the D–S evidence theory has been used in many fusion systems, and the creation of more efficient and rational basic probability assignments (BPAs) has been attempted. Liu et al. (2017) showed a human-machine autonomous system with a fuzzy decision-making fuser that fuses computer vision and human vision to detect targets in RSVP tasks. The fuzzy decision-making fuser uses a composite model that combines discriminative-type and generative-type methods to confirm basic BPAs. The major limitation of this approach is expensive computations, especially when BPAs are assigned to more than two heterogeneous information sources. Lee and Kwon (2021) proposed “task conversions” to fuse human and computer vision, in which a dynamic belief fusion (DBF) method is adopted that constructs precision-recall relationships as BPAs, and the probability distributions of all the heterogeneous information sources are fused using the D–S combination rule. However, the effect of using comprehensive indexes as a BPA function on performance improvement is relatively limited. As we all know, the quality of BPA directly affects classification performance after fusion (Wang and Tang, 2022). Therefore, construct a reliable BPA to improve the classification performance of the fusion methods remain a crucial problem.

In this study, we proposed a new D-S evidence theory fusion framework named dynamic probability integration (DPI), in which a new BPA approach was designed. This method was designed to solve the problem of no ERP components or miniature ERP components caused by the attention lapses of human vision in abnormal conditions, which is difficult for traditional algorithms to handle. To improve the classification performance, the detection performance models of target and non-target were constructed to assign probabilities for different heterogeneous information sources. The information from human and computer vision was obtained by the EEG-based BCI and YOLO V3 algorithm individually, and the EEG was decoded using a spatial-temporal hybrid common spatial pattern-principal component analysis (STHCP) algorithm. To evaluate the performance of the DPI in the abnormal condition, we applicated it to a nighttime vehicle detection task. The results prove that it is promising to be a general and effective target detection method in abnormal conditions. To the best of our knowledge, it is the first attempt to enhance the target detection performance of RSVP by fusing human and computer vision in an abnormal condition (nighttime).

We organized the rest of our paper as follows: section “Fusion method of computer vision and human vision based on dynamic probability integration” describes the fusion method of computer vision and human vision based on DPI. Section “Experiment settings” describes the design of the nighttime vehicle detection experiment. The experimental results are discussed in section “Results.” In section “Discussion,” we discuss the performance of DPI in different situations and the performance of STHCP with different numbers of training trials. Finally, Section VI contains the conclusions drawn.

## Fusion method of computer vision and human vision based on dynamic probability integration

The proposed method contains three parts, as follows: **(i)** obtaining computer vision information, **(ii)** obtaining human vision information, and **(iii)** dynamic probability integration. Let Θ represent a set of exhaustive and nonempty events in which elements are mutually exclusive, as indicated by the following:


(1) Θ={{target},{non⁢-⁢target}}

**FIGURE 1:**
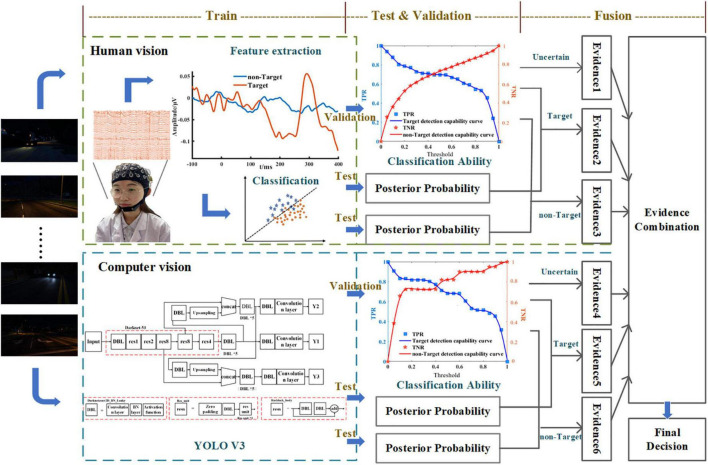
The process of dynamic probability integration (DPI).

Where {*target*} and {*non*-*target*}represents target and non-target images, respectively. The power set of Θ is 2^Θ^, and the 2^Θ^ elements are defined as follows


(2) $$2^{\Theta }=\left\{\emptyset ,\left\{\mathrm{target}\right\},\left\{\mathrm{non}\,\text{-}\,\mathrm{target}\right\},\left\{\mathrm{target},\mathrm{non}\,\text{-}\,\mathrm{target}\right\}\right\}$$

where the composite element represents uncertain information. The DPI process is shown in Figure 1. Two heterogeneous information source models were constructed to obtain the information from human vision and computer vision, respectively. The detection capability models of target and non-target from each heterogeneous information source were obtained. Posterior probabilities were estimated by each heterogeneous information source, representing the possibilities of targets and non-targets. The true positive rate (TPR) and true negative rate (TNR) combined with the posterior probabilities provided evidence for the three hypotheses (target, non-target and uncertain) (Zhu and Kan, 2022). Then, evidence was fused using the D–S combination rule.

### Computer vision

**FIGURE 2:**
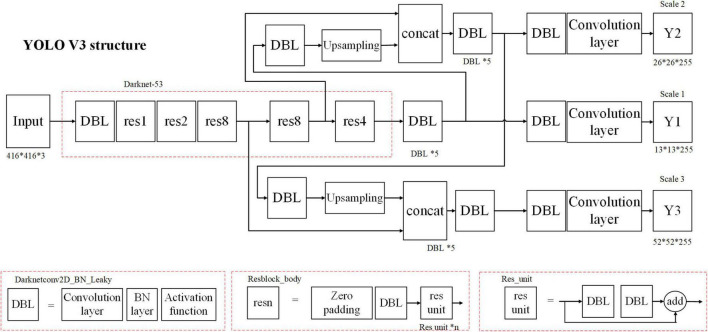
The network of YOLO V3. *Means multiplication.

The YOLO framework was proposed as a single regression problem, straight from image pixels to class posterior probability (Mao et al., 2020). Compared with YOLO, YOLO V3 improved the prediction accuracy on the premise of guaranteeing the advantage of detection speed. The network structure is shown in Figure 2. It uses a network structure named Darknet-53, consisting of only 53 convolutional layers. Five residual blocks are contained and each of them consists of multiple residual components, which are composed of convolutional layers and shortcut links. The YOLO V3 network has a deeper network structure through the residual network structure and a multi-scale detection method, which effectively detects large and small targets. We used YOLO V3 as a computer vision method for nighttime vehicle detection and obtained the posterior probabilities of targets and non-targets, which are defined as *p*_*t*_ and *p*_*nt*_, respectively. The training set contains two types of images: daytime vehicles and nighttime vehicles, with 1,000 images of daytime vehicles and 1,000 images of nighttime vehicles.

### Human vision

**FIGURE 3:**
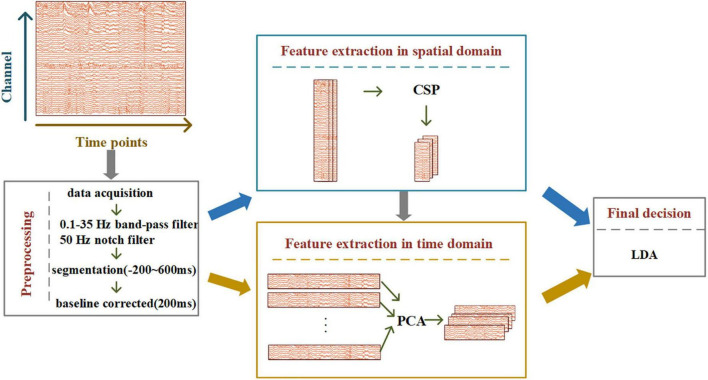
Flowchart of spatial-temporal hybrid common spatial pattern - principal component analysis (STHCP).

Inspired by HDCA and SWFP, we designed the STHCP algorithm, which uses common spatial pattern (CSP) and PCA to extract features in the spatial and temporal domains, respectively. The CSP can maximize the target state-related components (event-related potentials) and minimize the non-target state-related components (background EEG). The PCA can identify a hyperplane in the time domain. Finally, the spatial and temporal features of RSVP-BCI are constructed and then classified using a linear discrimination analysis. Figure 3 shows the specific STHCP process. The EEG signal processing consists of data preprocessing, feature extraction, and classification. In the preprocessing stage, the signal was notch filtered in 50 Hz, band-pass filtered between 0.1 and 35 Hz, the range of segmentation was between 200 and 600 ms, and baseline corrected with 200 ms before the stimulus. After that, the feature extraction process is as follows:

First, the CSP is used for spatial filtering. The covariance matrix of the two classes of data is as follows:


(3) $$\bar{U}=\frac{1}{n}\sum_{i=1}^{n}\frac{Y_{j}\,\left(i\right)\,Y_{j}^{T}\,\left(i\right)}{t\,r\,a\,c\,e\,\left(Y_{j}\,\left(i\right)\,Y_{j}^{T}\,\left(i\right)\right)}\left(j\in \left\{+,-\right\}\right)$$

Where *Y*_*i*_ ∈ *R^C^*^×*D*^ represents the *i*th trial of the EEG data after preprocessing, *C* represents the number of EEG channels, and *D* represents the length of time points per trial. *Y*_+_(*i*) and *Y*_−_(*i*) represent EEG data in a single trial of target and non-target states, respectively.

The CSP can be expressed as follows:


(4) $${\left\{\max,\min\right\}}_{W\in R^{j}}\,\frac{W^{T}\,{\bar{U}}_{+}\,W}{W^{T}\,{\bar{U}}_{-}\,W}$$

(5) $${\bar{U}}_{+}\,W=\lambda \,{\bar{U}}_{-}\,W$$

Where the spatial filter obtained by CSP is denoted as $W\in R^{C\times \bar{C}}$, in which $\bar{C}$ represents the number of spatial filters selected by the CSP. After the CSP, EEG data are defined as follows:


(6) $$Y_{C\,S\,P}=W^{T}\,Y_{C\,S\,P}$$

Second, the PCA is used to reduce the time domain dimension. The data of each channel of *Y*_*CSP*_ are reduced using the PCA, as follows:


(7) $$V=\frac{{\bar{Y}}_{C\,S\,P}\,{\bar{Y}}_{C\,S\,P}^{T}}{n-1}$$

Where *V* represents the covariance matrix of ${\bar{Y}}_{C\,S\,P}$, and *n* represents the number of samples. The eigenvalues and corresponding eigenvectors of *V* are calculated, and then, the first *K* principal components are selected to represent the domain information of each channel. The corresponding eigenvectors *X* is a projection matrix for feature extraction, as follows:


(8) $$Z=Y^{C\,S\,P}\,X$$

Where *Z*_*i*_ represents the obtained feature.

Finally, *Z*_*i*_ is classified by linear discrimination analysis classifier to the posterior probability of the target, as follows:


(9) $$p_{t}=f\,\left(Z\right)$$

The posterior probability of non-target is (1−*p*_*t*_). Referring to relevant research results (Müller-Gerking et al., 1999; Blankertz et al., 2011), we set the range of parameters to shorten the training time, where $\bar{C}\in \left[2,10\right]$, *K* ∈ [1,10].

### Dynamic probability integration

**FIGURE 4:**
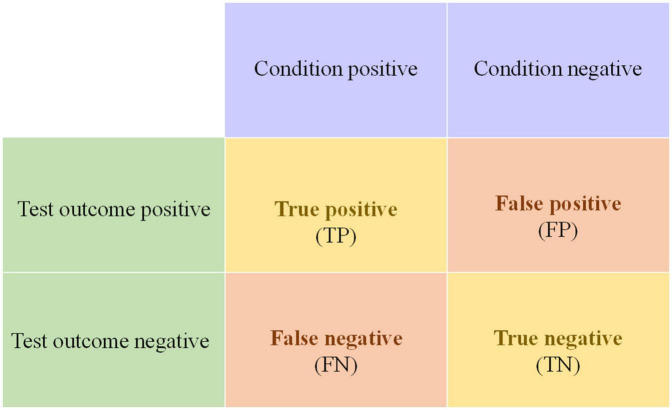
Confusion matrix.

The probabilities were assigned to all the hypotheses in accordance with the detection performances of targets and non-targets for each heterogeneous information source. The TPR and TNR represent the detection capabilities of targets and non-targets, respectively. In addition, the false positive rate (FPR) is another performance metric that refers to the probability of falsely identifying the non-target as the target (Zhu and Kan, 2022). The above indicators are calculated from the confusion matrix, and an illustration of the confusion matrix is shown in Figure 4.

**FIGURE 5:**
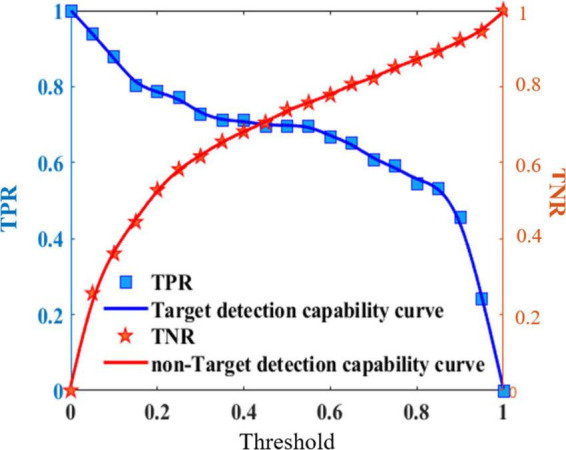
Detection performance curve of heterogeneous information source.

The detection performance can be obtained using different threshold settings. Thus, a detection performance model can be built for each heterogeneous information source. Figure 5 shows the detection performance curve of a heterogeneous information source.

The samples were divided into three parts, namely training, validation, and testing sets, and the proportions of the three parts were 2:1:1. A fourfold cross-validation scheme was used to ensure that all the methods were trained, verified, and tested on independent data. The data were randomly divided into four equally sized blocks, with two blocks selected as the training set, one as the validation set, and one as the testing set. This process was repeated four times, and the average was used as the result. Both human and computer vision models were constructed using the training set. Human and computer vision were two heterogeneous information sources, indicated by φ_1_ and φ_2_, respectively. Five steps were used to classify the images in the testing set using DPI.

In step 1, we used the validation set to evaluate the detection performance of each heterogeneous information source. We used the Smoothing function in the curve fitting of the MATLAB toolbox to construct a detection performance model on the basis of the TPR-TNR relationship curve. The target detection performance model of heterogeneous information source φ was assumed to be *g*^φ^(*x*), and the non-target detection performance model was *f*^φ^(*x*).

In step 2, the threshold *t*_best_ which maximized the difference between TPR and FPR was determined in the validation set.

In step 3, the probabilities of the samples that were predicted as target and non-target by heterogeneous information source φ in the testing dataset were defined as $p_{t}^{\varphi }$ and $p_{n\,t}^{\varphi }$, respectively. Then, the evidence obtained by φ can be defined as follows:


(10) $$m^{\varphi }\,\left(\text{target}\right)=g^{\varphi }\,\left(t_{\text{best}}\right)\times p_{t}^{\varphi }$$

(11) $$m^{\varphi }\,\left(\mathrm{non}\,\text{-}\,\mathrm{target}\right)=f^{\varphi }\,\left(t_{\text{best}}\right)\times p_{n\,t}^{\varphi }$$

(12) $$m^{\varphi }\,\left(\left\{\mathrm{target},\mathrm{non}\,\text{-}\,\mathrm{target}\right\}\right)=1-m^{\varphi }\,\left(\text{target}\right)-m^{\varphi }\,\left(\mathrm{non}\,\text{-}\,\mathrm{target}\right)$$

Where *m*^φ^(target) represents evidence of the target, *m*^φ^(*non*-*target*) represents evidence of the non-target, and *m*^φ^({target,*non*-*target*}) represents evidence of uncertain information.

In step 4, the combined results were obtained using the evidence provided by different heterogeneous information sources. Assuming that *m*(A) represents the evidence that supports hypothesis A, the evidence for different hypothesis were combined using the D–S combination rule “⊕” which was defined as follows:


(13) $$m^{\varphi_{1}}⊕m^{\varphi_{2}}\,\left(\text{A}\right)=\left\{ \begin{array}{cc} \frac{1}{1-K}\,\sum_{\text{B}\cap \text{C}=\text{A}}m^{\varphi_{1}}\,\left(\text{B}\right)\,m^{\varphi_{2}}\,\left(\text{C}\right), & \text{A}\neq \emptyset \\ 0, & \text{A}=\emptyset \end{array}\right.$$

(14) $$\text{K}=\sum_{\text{B}\cap \text{C}=\emptyset }m^{\varphi_{1}}\,\left(\text{B}\right)\,m^{\varphi_{2}}\,\left(\text{C}\right)$$

Where *K* represents conflicts among the evidence, called the conflict coefficient between *B*and *C*. *A*, *B* and C∈2^Θ^.

For convenience, let “uncertain” represent {target, non-target}. Then, the combination results for the three hypotheses could be expressed as follows:


(15) $$m\,\left(\text{target}\right)=m^{\varphi_{1}}\,\left(\text{target}\right)⊕m^{\varphi_{2}}\,\left(\text{target}\right)$$

(16) $$m\,\left(\text{nontarget}\right)=m^{\varphi_{1}}\,\left(\mathrm{non}\,\text{-}\,\mathrm{target}\right)⊕m^{\varphi_{2}}\,\left(\mathrm{non}\,\text{-}\,\mathrm{target}\right)$$

(17) m⁢(uncertain)=1-m⁢(target)-m⁢(nontarget)

Finally, the predicted classes were determined based on the pignistic transformation: if *P*_*pig*_ < *t*_*best*_, then *class* = 0that represents this sample is the non-target, whereas if *P*_*pig*_ > *t*_*best*_, *class* = 1that represents this sample is the target. The best threshold was determined by the validation dataset, then used to evaluate performance on the testing set.


(18) $$P_{p\,i\,g}\,\left(\text{target}\right)=m\,\left(\text{target}\right)+m\,\left(\text{uncertain}\right)$$

(19) $$P_{p\,i\,g}\,\left(\text{nontarget}\right)=m\,\left(\text{nontarget}\right)+m\,\left(\text{uncertain}\right)$$

Our proposed DPI has three merits. Firstly, DPI has inherent advantages in handling and expressing uncertain information compared with other algorithms based on probabilities. Secondly, DPI can extract complementary information from both computer and human vision to ensure classification performance. Thirdly, DPI thoroughly considered the detection capacity of targets and non-targets from each heterogeneous information source and applied it to the probability assignment strategy, which can further improve the overall classification performance.

## Experiment settings

An RSVP experiment was conducted to verify the performance of DPI and STHCP in nighttime vehicle detection.

### Participants

Nine subjects with no history of psychiatric or neurological problems were recruited to participate in the RSVP experiment. Three of them were female, they were all right-handed, and their ages ranged from 21 to 24. All the subjects had the normal or corrected-to-normal vision, and four out of the nine subjects had previous experiences with BCI. Before participation, all the subjects were required to sign an informed consent. All the experimental procedures were approved by the Northwestern Polytechnical University Medical and Experimental Animal Ethics Committee.

### Rapid serial visual presentation protocol

**FIGURE 6:**
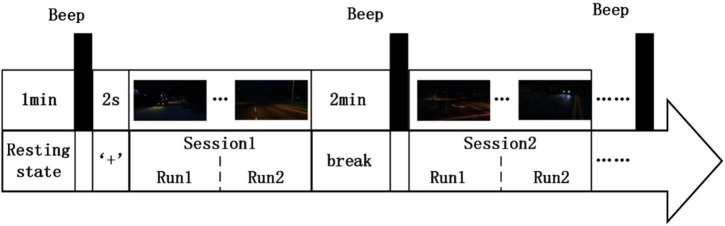
Process of RSVP paradigm.

Subjects sat in a suitable chair in front of a screen. The experiment began with a 1-min resting state, and a beep sounded at the end of the resting state. Then, a “+” fixation was displayed on the screen for 2s to correct the visual position. The RSVP paradigm process contained two sessions, and each session contained two runs. There was a 2-min break between the two sessions. The RSVP paradigm process is shown in Figure 6. The subjects were asked to respond to target images by pressing the blank space key.

**FIGURE 7:**
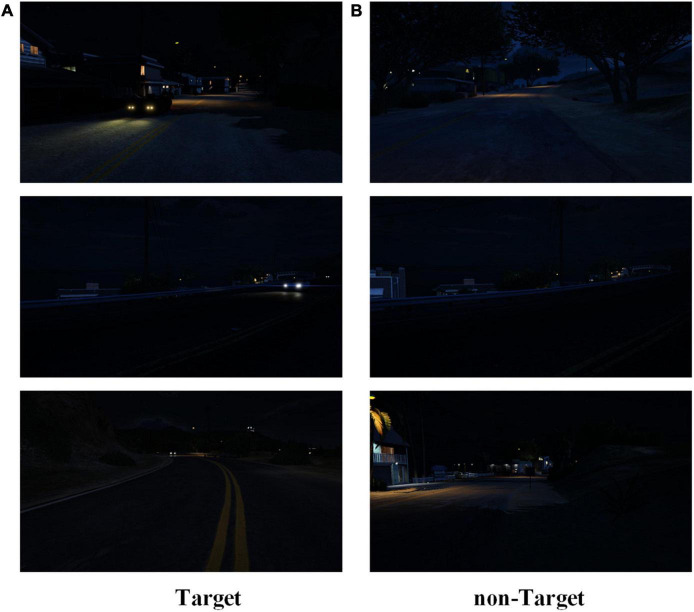
Night driving demonstration. **(A)** Target images demonstration in the nighttime condition. **(B)** Non-target images demonstration in the nighttime condition.

The image stream included 1,500 target and non-target images, with the target image proportion being 25%. Each image was presented randomly on the screen for 200 ms. The night driving demonstration is shown in Figure 7.

### Data acquisition

EEG signals were recorded by a Neuracle wireless amplifier from international 10–20 EEG caps having 64 electrodes located over the scalp region. All the channels were referenced to the Cz electrode, which was placed in the central-parietal area.

### Evaluation

The data used was highly unbalanced, and accuracy is sensitive to the sample proportion, making it inappropriate as an evaluation indicator. Therefore, the performance was evaluated using the area under the receiver operating characteristic curve (AUC), TPR, and FPR. In addition to these indicators, balanced accuracy (BA), which gets the balance between TPR and FPR, was used as a performance indicator in this study (Yu et al., 2011; Teng et al., 2018). In conclusion, four indicators (TPR, FPR, AUC, and BA) were used in this paper, among which AUC and BA are the comprehensive indicators for performance evaluation.

## Results

### Analysis of event-related potentials components

**FIGURE 8:**
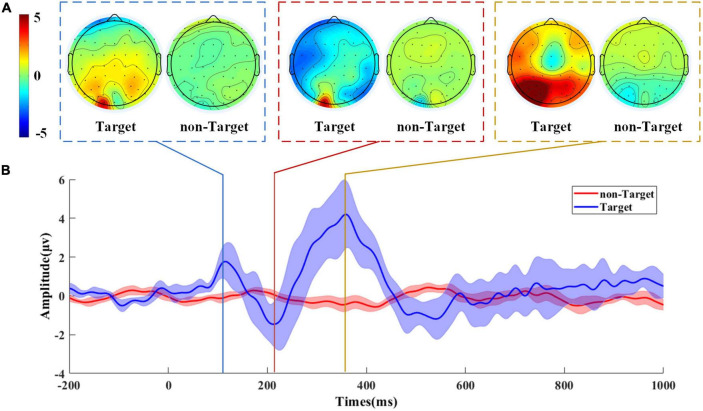
The grand-average ERP waveform and topographies of target and non-target. **(A)** The grand-average topographies at the peak of ERP waveform. The topography of the target is on the left, and on the right is the topography of the non-target. **(B)** The grand-average ERP waveform of target and non-target from the Pz channel. The purple line represents the target waveform, and the orange line is the non-target waveform. The standard deviation in the grand-average ERP waveforms is represented as shaded regions.

Figure 8 shows the grand-average ERP waveforms from the Pz channel and topographies at the peak of the ERP waveform to verify the ERP components evoked by the nighttime vehicle detection task. As can be seen, it is significant that the grand-average ERP waveform and topographies under the target and non-target are different. P1, N2, and P3 can be found at the time window of 100–150, 150–250, and 350–400 ms, which indicate attention allocation, stimulus discrimination, and attention processing, respectively. The topographies show that the P1 component is dominant over the lateral occipital areas, the N2 component appears over most brain regions, and the P3 component can be observed over the central-parietal areas.

### Estimating performance from electroencephalography

**FIGURE 9:**
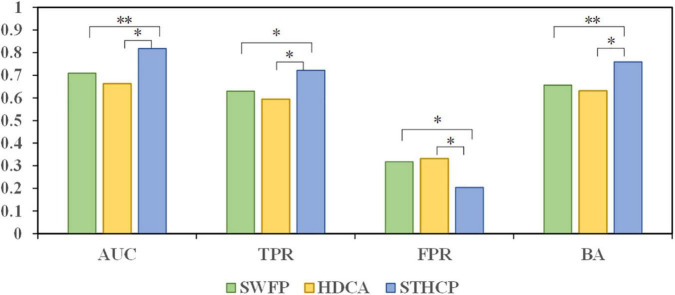
The classification results compared to our proposed STHCP, HDCA, and SWFP. “*” indicates a significant difference (Wilcoxon signed rank test) between STHCP and other approaches: * and ^**^ means *p* < 0.05 and *p* < 0.01.

We compared the classification performance of the proposed STHCP with HDCA and SWFP, and the results are depicted in Figure 9. The Wilcoxon signed rank test was adopted to estimate the significant differences among these approaches, “*” and “^**^” indicate the significate level. STHCP obtained an average AUC of 0.818 ± 0.06, 15.4% higher than that of SWFP and 23.4% higher than that of HDCA. An average TPR of STHCP achieved 0.722 ± 0.056, 14.6% higher than that of SWFP and 21.5% higher than that of HDCA. An average BA of STHCP achieved 0.760 ± 0.007, which was 15.9% higher than that of SWFP and 20.4% higher than that of HDCA. STHCP obtained an average FPR of 0.204 ± 0.093, which was 35.6% lower than that of SWFP and 38.6% lower than that of HDCA. Among the three approaches, STHCP obtained the highest AUC, TPR, BA, and the lowest FPR.

**FIGURE 10:**
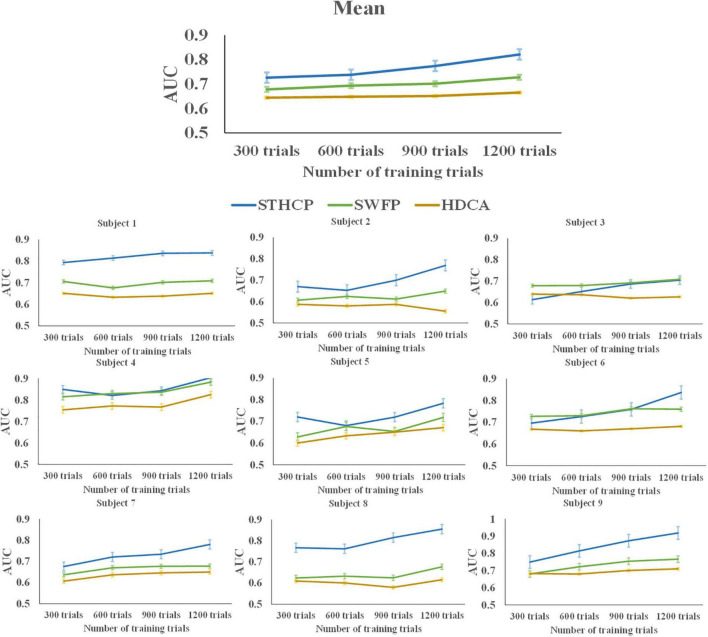
The AUC of STHCP, SWFP, and HDCA with different training trials.

The AUC performance of STHCP, SWFP, and HDCA with the training trials ranging from 300 to 1,200 was estimated, and the results are shown in Figure 10. As a result, the mean AUCs of the three methods were improved with the increasing number of the training trials.

### Performance comparison between dynamic probability integration and other fusion approaches

The average TPR, FPR, BA, and AUC values for the nine subjects are shown in Table 1. Two state-of-the-art fusion algorithms (DBF and NBF) and two individual heterogeneous information source decoding methods were compared with DPI. The Wilcoxon signed rank test was conducted to analyze the significant differences between DPI and the other algorithms. The TPR, AUC, and BA of the proposed DPI were significantly better than those of the other four algorithms. Specifically, the average AUC of DPI reached 0.912 ± 0.041, representing increases of 11.5, 5.2, 1.7, and 3.4% compared with human vision, computer vision, DBF, and NBF, respectively. The average TPR of the proposed DPI reached 0.810 ± 0.068, representing increases of 12.2, 21.8, 6.6, and 28.6% compared with human vision, computer vision, DBF and NBF, respectively. The average FPR of DPI was higher than NBF, but smaller than those of human vision, computer vision and DBF. Congruently, the BA of DPI was significantly superior to human vision, computer vision, DBF, and NBF, showing increases of 11.3, 10.2, 3.8, and 7.1%.

**TABLE 1:** Mean and standard deviation of evaluation measurements for 9 subjects.

	TPR	FPR	AUC	BA
Human vision	0.722 ± 0.056**	0.204 ± 0.093	0.818 ± 0.060**	0.759 ± 0.007**
Computer vision	0.665 ± 0.154**	0.131 ± 0.100	0.867 ± 0.028*	0.767 ± 0.069**
DBF	0.760 ± 0.082	0.135 ± 0.142	0.897 ± 0.040**	0.813 ± 0.050**
NBF	0.630 ± 0.060**	**0.052 ± 0.056**	0.882 ± 0.047**	0.789 ± 0.038**
DPI	**0.810 ± 0.068**	0.120 ± 0.074	**0.912 ± 0.041**	**0.845 ± 0.052**

DBF, dynamic belief fusion; NBF, naive Bayesian fusion; DPI, dynamic probability integration. “*” indicates significant difference (Wilcoxon signed rank test) between DPI and other approaches: * and ** means *p* < 0.05 and *p* < 0.01. The best performance is shown in bold face.

**FIGURE 11:**
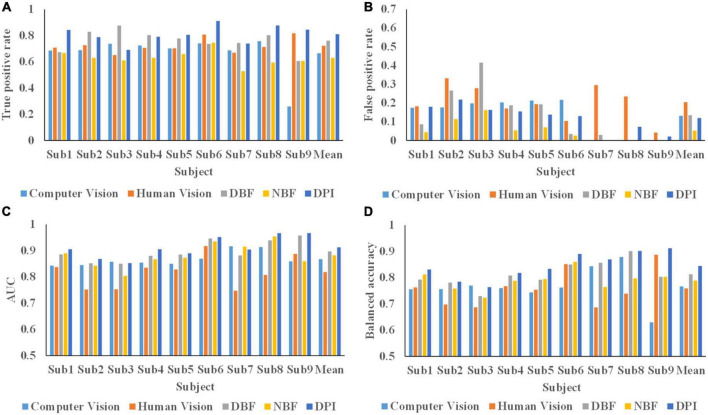
Classification performance of 9 subjects. **(A)** True positive rate of 9 subjects in all methods. **(B)** False positive rate of 9 subjects in all methods. **(C)** AUC value of 9 subjects in all methods. **(D)** Balanced accuracy of 9 subjects in all methods.

The analysis of TPR, FPR, AUC, and BA in nine subjects are depicted in Figure 11. The AUCs for the DPI of six subjects were greater than 0.9. The AUCs of DPI were significantly improved in most subjects, except for subjects 3 and 7. Although the AUC of DPI in subject 3 was slightly lower than that of computer vision, it was superior to the other fusion methods. For subject 7, the AUC of DPI was smaller than those of computer vision and NBF, but the BA, TPR, and FPR of DPI were superior to those of the other methods. The BAs for the DPI for seven subjects were greater than 0.8. Specifically, the BA of the DPI was significantly better than those of computer vision, human vision, and other fusion methods, except for that of subject 3. Although the BA of DPI for subject 3 was slightly lower than that of computer vision, it was higher than those of human vision and other fusion methods. While the classification performance varied across subjects, DPI showed a better performance than human vision, computer vision, and two fusion methods.

## Discussion

In this article, the STHCP and DPI were proposed for enhancing the performance of the nighttime vehicle detection RSVP task. It is difficult for RSVP-BCI to be applied in the real world, owing to its erratic performance. In particular, the problem that no feature or miniature feature is evoked caused by attention lapse in some abnormal conditions is difficult to be solved by the existing methods. Experimental results show that the highest AUC from HDCA and SWFP in the nighttime condition is lower than that of the method in some normal environments (Alpert et al., 2014; Marathe et al., 2014; Song et al., 2021), which remind us to map out the specific strategy to solve this problem. Our proposed DPI can extract complementary information from two heterogeneous information sources to solve the above problem. The experimental results illustrate that DPI got the highest average AUC (0.912 ± 0.041) compared with two individual information source classifiers and two excellent fusion methods. Furthermore, DPI achieved the highest BA (0.845 ± 0.052) by a novel probability assignment strategy that comprehensively considers the classification performance of individual information source for target and non-target. Our proposed methods can promote the development of RSVP-BCI to apply in the real world. To better demonstrate the classification performance of our proposed methods, the following analyses were made.

### Performance of spatial pattern-principal component analysis with different number of training trials

The AUC of STHCP, SWFP, and HDCA with different training trials is shown in Figure 10. The AUCs of STHCP are higher than the other two methods in all subjects except for subject 3 and subject 4, regardless of the number of training trials. Specifically, STHCP outperformed SWFP only when the number of training trials increased to 1,200 in subject 3. In subject 4, the AUC of STHCP is lower than SWFP only when the number of training trials is 600. Compared with SWFP and HDCA, the classification performance of STHCP is more sensitive to the sizer of the training set. When the number of training trials increases, the performance will significantly improve.

### Performance of dynamic probability integration in conflict and non-conflict situations

According to recent studies, the performance of fusion methods may be limited when decision conflict occurs (Deng et al., 2016; Li and Xiao, 2021). Large differences in classification performances between the two heterogeneous information sources may lead to decision conflicts. In order to discuss the decision conflicts, the difference in classification performance was defined as the difference between AUC values. The probability of decision conflict may be improved when the difference between AUC values is close to or above 0.1. The AUC and BA of all the methods in conflict and non-conflict situations are shown in Table 2. Subjects 1, 4, 5, 6, and 9 belonged to the non-conflict situation. The AUCs of all fusion methods are higher than that of individual information source in the non-conflict situation. Specifically, the AUC and BA of DPI in the non-conflict situation were better than those of other methods, and the AUC of 4 out of 5 subjects was greater than 0.9. These indicated that DPI is the best-performing method in the non-conflict situation. Subject 2, 3, 7, and 8 belonged to the conflict situation. Note that AUCs of two baseline fusion methods in 3 out of 4 subjects are lower than computer vision which is contrary to the results in non-conflict situations. Subject 2 and 8 had better AUC and BA in the conflict situation using DPI than the other methods. Although the AUC and BA of DPI were slightly lower than that of computer vision in subject 3, it had a better performance than the other fusion methods. The AUC of DPI in subject 7 was smaller than that of computer vision, but the BA of DPI was higher than those of the other methods. The data indicate that although conflict situations limit the performances of most fusion methods, DPI was still superior to the other fusion methods.

**TABLE 2:** The classification performance in conflict and non-conflict situations.

		Non-conflict					Conflict			
			
		s1	s4	s5	s6	s9	s2	s3	s7	s8
Computer vision	AUC	0.843	0.854	0.850	0.870	0.859	0.845	**0.857**	**0.916**	0.914
	BA	0.756	0.76	0.744	0.762	0.63	0.756	**0.770**	0.843	0.879
Human vision	AUC	0.837	0.835	0.828	0.917	0.887	0.752	0.753	0.747	0.807
	BA	0.763	0.767	0.754	0.851	0.888	0.697	0.686	0.686	0.739
DBF	AUC	0.886	0.88	0.885	0.946	0.958	0.852	0.850	0.881	0.939
	BA	0.793	0.808	0.792	0.850	0.803	0.781	0.730	0.857	0.901
NBF	AUC	0.89	0.867	0.873	0.935	0.859	0.842	0.804	0.915	0.954
	BA	0.812	0.788	0.795	0.860	0.803	0.758	0.724	0.764	0.797
DPI	AUC	**0.905**	**0.905**	**0.890**	**0.952**	**0.967**	**0.868**	0.852	0.905	**0.966**
	BA	**0.831**	**0.818**	**0.834**	**0.890**	**0.912**	**0.784**	0.764	**0.869**	**0.902**

DBF, dynamic belief fusion; NBF, naive Bayesian fusion; DPI, dynamic probability integration. The best performance is shown in bold face.

There are several limitations in the present study. First, only nine subjects were included, and further studies should expand the dataset. Second, no specific strategy was proposed for decision conflict in DPI. Even though the DPI performance was better than other fusion methods, a special strategy should be to promote the performance in conflict situations. Third, several types of RSVP, such as dual-RSVP and triple-RSVP, should be adopted to evaluate our proposed method.

## Conclusion

To solve the problem of no ERP components or miniature ERP components caused by the attention lapses of human vision in abnormal conditions, we proposed a human-computer vision fusion method named DPI. Moreover, a spatial-temporal hybrid feature extraction method was included to decode RSVP-EEG signals. Two heterogeneous information source models, RSVP-based BCI and YOLO V3, were constructed to obtain the detection performance model and posterior probabilities. Then, a new BPA function assigned weights to different heterogeneous information sources by constructing the detection performance model, which fully considers the detection accuracy of target and non-target. Afterward, the D–S combination rule fused the two heterogeneous information sources. We recruited nine subjects to participate in a nighttime vehicle detection experiment to evaluate the performance of DPI in attention lapses. The experimental results showed that STHCP obtained the highest AUC of 0.818 ± 0.06 compared with the other two baseline methods and increased by 15.4 and 23.4%. The average AUC of the proposed DPI reached 0.912 ± 0.041, which increased by 11.5 and 5.2% compared with human vision and computer vision, respectively, with increases of 3.4 and 1.7% compared with NBF, and DBF, respectively. The BA of the proposed method was better than those of human vision, computer vision, NBF and DBF, showing increases of 11.3, 10.2, 7.1, and 3.8%. As a result, DPI had the best performance compared with human and computer vision methods and two excellent fusion methods. Our study provides a new direction for RSVP task performance improvement and promotes the development of the practical process in RSVP-BCI.

## Data availability statement

The raw data supporting the conclusions of this article will be made available by the authors, without undue reservation.

## Ethics statement

The studies involving human participants were reviewed and approved by Northwestern Polytechnical University Medical and Experimental Animal Ethics Committee. The patients/participants provided their written informed consent to participate in this study.

## Author contributions

YC developed the dynamic probability integration method and designed the nighttime vehicle detection experiment. SX and XX made the major contribution in supervised the research and helped in finalizing the manuscript. XZ and XL assisted with data collection and analysis. All authors read and approved the final manuscript.
